# GENERATIVITY, COGNITIVE REAPPRAISAL, AND OPTIMIZING SELF-ACCEPTANCE ACROSS MID LIFE AND OLD AGE

**DOI:** 10.1093/geroni/igad104.2688

**Published:** 2023-12-21

**Authors:** Madeline Nichols, John Geldhof, Robert Stawski

**Affiliations:** Oregon State University, Corvallis, Oregon, United States; Oregon State University, Corvallis, Oregon, United States; University of Essex, Colchester, England, United Kingdom

## Abstract

Midlife is an opportune time during which individuals can establish patterns of behavior (e.g., generativity) and self-regulatory processes (e.g., emotion regulation) that will optimize their psychological wellbeing and aging. We sought to test whether: 1.) generativity is directly associated with better self-acceptance, 2.) the effect of generativity is indirect and mediated by emotion regulation (i.e., cognitive reappraisal), and 3.) age moderates associations among generativity, cognitive reappraisal, and self-acceptance. Using data from the second and third waves of the Midlife in the United States (MIDUS) study (N=935, Mage=54.36, SD=11.05, Range = 34-83, 55.08% Female), results revealed that cognitive reappraisal (β= 0.07, p< .01) and older age (β= 0.06, p= .02) were associated with higher self-acceptance; however, generativity was not, and age did not predict generativity nor cognitive reappraisal. Generativity was associated with more frequent use of cognitive reappraisal (β= 0.16, p< .001), with cognitive reappraisal then significantly mediating the association between generativity and self-acceptance (Natural Indirect Effect= 0.02, SE= .01, p= .02). Age significantly interacted with generativity when predicting self-acceptance (β= 0.39, p= .02), such that the effect of generativity increased with age. These findings are important for reframing midlife as a critical developmental stage during which individuals can engage in behaviors and self-regulatory processes that optimize their self-acceptance throughout the aging process. As age did not moderate most associations, interventions could target generativity to promote effective emotion regulation and cognitive reappraisal to enhance self-acceptance across adulthood and into later life given its positive implications for wellbeing, health, and longevity.

